# Autoimmune thyroiditis and celiac disease do not worsen endothelial function in subjects with type 1 diabetes: an observational study

**DOI:** 10.1186/s13098-022-00877-y

**Published:** 2022-07-23

**Authors:** Martina Parise, Antonio Cutruzzolà, Faustina Barbara Scavelli, Claudio Carallo, Agostino Gnasso, Concetta Irace

**Affiliations:** 1 Department of Health Science, University Magna Græcia, Viale Europa, Località Germaneto, Catanzaro, Italy; 2 Department of Clinical and Experimental Medicine, University Magna Græcia, Catanzaro, Italy; 3 Azienda Ospedaliero-Universitaria Mater Domini, Catanzaro, Italy

**Keywords:** Type 1 diabetes, Autoimmune thyroiditis, Coeliac disease, Atherosclerosis, Endothelial function, Reactive hyperemia flow mediated dilation

## Abstract

**Background:**

Type 1 diabetes (T1D) is frequently associated with autoimmune thyroiditis (AT) and coeliac disease (CD). Whether the coexistence of multiple autoimmune diseases increases cardiovascular risk is uncertain. We evaluated the effects of AT and CD on arterial wall thickening and endothelial function in patients with T1D.

**Methods:**

This observational study analyzed data from T1D patients regularly followed by the Diabetes Care Centre. Clinical and biochemical characteristics and micro and macrovascular complications were collected from the electronic medical records. All subjects performed Echo-Doppler to evaluate Intima-Media Thickness (IMT) of the common carotid artery (CCA) and endothelial function by the flow-mediated dilation (FMD) technique. The statistical analyses were performed by SPSS for Macintosh. Comparison between means was performed using the t-test for unpaired data and the Mann–Whitney U test. The ANalysis Of VAriance and the Tukey posthoc test were applied to compare patients with and without other autoimmune diseases, and control subjects. The p-value for statistical significance was set at p < 0.05.

**Results:**

A total of 110 patients were enrolled. Among these, 69 had T1D and 41 T1D and AT and or CD, of whom 33 AT, 7 CD, and 1 both AT and CD. The mean age was 35 years, mean HbA1c was 7.6%, and mean diabetes duration 18 years. The IMT of the CCA was not significantly different between T1D patients with and without concomitant autoimmune diseases (with AT and CD: right CCA 603 ± 186 µ, left 635 ± 175 µ; without AT and CD: right CCA 611 ± 176 µ, left CCA 631 ± 200 µ). FMD was also comparable between T1D groups, with AT and CD 7.9 ± 4.2%; without AT and CD 8.8 ± 4.4%.

**Conclusion:**

Patients with T1D and concomitant AT and or CD show no worse morphological or functional vascular damage, evaluated by CCA IMT and brachial artery flow-mediated dilation, than patients with T1D alone.

## Introduction

Type 1 diabetes (T1D) is an autoimmune disease accounting for approximately 5–10% of all forms of diabetes [1]. Despite improvements in therapy, T1D is still burdened by significant excess morbidity and mortality, compared to the general population, mainly due to a higher incidence of macroangiopathy [2]. As is often observed in autoimmune diseases, T1D is frequently accompanied by other diseases that share the same pathogenetic mechanism, notably autoimmune thyroiditis (AT) and coeliac disease (CD). The prevalence of AT and CD amounts to 25% and 4% in T1D patients, respectively. At the same time, other manifestations such as Addison's disease, rheumatoid arthritis, pernicious anemia, and chronic inflammatory bowel disease are less common [3]. Overall, autoimmune diseases are more prevalent in women, increasing with age [4]. A recent prospective study including about 180,000 females and males from the US with T1D showed that the coexistence of multiple autoimmune diseases is associated with a greater frequency of renal failure, ischemic stroke, and myocardial infarction [5].

Furthermore, the greater the number of autoimmune diseases and the greater the risk to develop life-threatening complications. The type of autoimmune disease could also affect the entity of the risk. The risk of stroke and myocardial infarction is 1.63 and 1.46 in T1D with AT and 1.28 and 1.33 in T1D with CD. The molecular mechanisms underlying this synergy are not yet known, although the chronic inflammation that characterizes autoimmune diseases has been partly held responsible [6, 7]. From this perspective, patients with concomitant autoimmune diseases should receive a more intensive multifactorial treatment to prevent acute clinical events.

In this contest, the markers of early atherosclerosis such as endothelial dysfunction and arterial wall thickening might help identify patients who need more intensive intervention and more frequent monitoring of their cardiovascular risk factors.

Unfortunately, there are few and often contrasting results about the effect of AT and CD on the development of early atherosclerosis in patients with T1D. Some small studies have demonstrated that AT and CD affects carotid intima-media thickness (IMT) [8, 9], arterial stiffness [10], carotid pulsatility [11], and microvascular abnormalities [12, 13]. Conversely, the endothelial function seems to be not blunted by the coexistence of T1D and AT [10]. Other authors have hypothesized a possible protective effect of CD on prothrombotic state and the development of microvascular complications [14]. Considering this scenario, we have designed our study to evaluate if the co-occurrence of AT and or CD is associated with arterial wall thickening and endothelial dysfunction in patients with T1D.

## Patients and methods

Patients with T1D and with or without concomitant AT and or CD were recruited among those regularly followed at the Hospital Diabetes Care Centre, University Magna Græcia, Catanzaro. The study hypothesized that the co-presence of multiple autoimmune diseases causes increased endothelial dysfunction. Therefore, the sample size was calculated assuming at least a 30% difference in FMD between patients with only T1D and those with concomitant autoimmune diseases (mean FMD 8.2 ± 4.0%, α = 0.8, β = 0.05, n = 42 in each group). Starting in March 2021, patients returning for follow-up at the Diabetes Care Centre were consecutively enrolled. After 6 months, having reached the number of 41 in the group of T1D patients with concomitant AT and or CD, the enrolment was closed, and the data were analyzed. The upcoming COVID-19 outbreak wave prompted the decision to end the enrolment. Thirty-five healthy subjects, comparable in age and sex to all patients with T1D, were enrolled simultaneously among students and hospital staff as a control group for vascular data.

The following clinical and anthropometric parameters were taken from the electronic medical record or collected at the time of vascular study: age, gender, disease duration, age of onset of diabetes, glycated hemoglobin (HbA1c), fasting plasma glucose (FPG), body weight, height, waist circumference, body mass index (BMI) calculated as kg/m^2^, lipids, blood pressure, heart rate (HR), insulin strategy treatment (CSII, continuous subcutaneous insulin infusion; MDI multiple daily insulin injections), glucose monitoring (CGM, continuous glucose monitoring; SMBG self-monitoring blood glucose) micro- and macrovascular complications. Patients with T1D are yearly screened for long-term complications. AT and CD are generally looked for at the diagnosis of diabetes or at any time if specific symptoms occur. The diagnosis of AT was based on anti-thyroid peroxidase (TPO) antibodies. The CD diagnosis was based on autoantibodies to tissue transglutaminase and histological evidence of villous abnormalities in duodenal biopsy samples [15].

For the aim of our study, all subjects performed an Echo-Doppler examination to evaluate IMT of common carotid arteries and endothelial function by the flow-mediated dilation (FMD) technique. The local Ethical Committee approved the protocol. All subjects gave informed consent for the vascular study and data collection from the electronic files.

The vascular study was performed by an expert sonographer using the Echo-Doppler Philips HD 11XE (Royal Philips Electronics, the Netherlands) equipped with a multi-frequency 12–7 MHz linear transducer.

Carotid IMT was measured offline by dedicated software (AUTODESK Design Review, BSA, Italy, https://knowledge.autodesk.com/it) at the far wall of the common carotid artery, 1 cm proximal to the bulb, in three different projections, anterior, later, and posterior, and the mean value calculated. IMT was defined as the distance between the leading edge of the lumen-intima interface and the leading edge of the media-adventitia interface.

Endothelial function was evaluated at the brachial artery of the non-dominant artery by the widely used flow-mediated technique (FMD), which assesses the percentage of vasodilation of the brachial artery after ischemia. The ischemia is induced by a pneumatic cuff placed around the forearm and inflated to a supra-systolic pressure. After the ischemic stimulus is removed, brachial artery blood flow velocity increases, stimulating the endothelial cell to release nitric oxide, which induces arterial dilation. The percentage increase of brachial artery internal diameter (ID) estimates endothelial function. Brachial artery ID is defined as the distance between the leading edge of the intima lumen interface of the near wall and the leading edge of the lumen-intima interface of the far wall. We measured ID at three different locations of the brachial artery 1 cm proximal to the elbow and the mean value used for the analyses. FMD was calculated by the following formula: post-deflation ID—baseline ID/baseline ID. ID was measured offline at baseline and 1 min after 5 min of forearm ischemia, as suggested by the current guidelines.

The statistical analyses were performed by SPSS for Macintosh. Patients were divided in three different groups, those with T1D and AT and or CD, those with T1D and without AT or CD, and control group. Variables not normally distributed were triglycerides and SBP. Comparison between means was performed using the *t*-test for unpaired data and the Mann–Whitney U test. The ANOVA (ANalysis Of VAriance) and the Tukey posthoc test were applied to compare patients divided into those with T1D and other autoimmune diseases (AT and or CD), those with T1D and without other autoimmune diseases (AT and or CD), and control subjects, and to compare patients with T1D and without other autoimmune diseases (AT and or CD), patients with T1D and only AT, and patients with T1D and only CD. The p-value for statistical significance was set at p < 0.05.

## Results

A total of 110 patients were recruited for the study. Sixty-nine had T1D, and 41 had T1D with AT and or CD, of whom 33 had AT, 7 CD, and 1 had both diseases. All patients with CD were on a gluten-free diet, and 13 with AT were on hormone replacement therapy. Table 1 displays the clinical characteristics, co-morbidities, and complications of the two groups. The prevalence of females, hyperlipidemia, and CSII users was higher in patients with AT and or CD.

**Table 1:** Clinical and biochemical characteristics of T1D patients

Variable	T1D without AT and CD	T1D with AT and or CD	*p*
Number	69	41	–
Age (y)	38 ± 13	35 ± 14	0.34
Gender (Female %)	39	61	**0.02**
Disease duration (y)	16 ± 9	18 ± 10	0.33
Age at the onset of diabetes (y)	21 ± 13	16 ± 12	0.09
HbA1c (%)	7.8 ± 1.2	7.6 ± 0.8	0.34
FPG (mg/dL)	162 ± 68	136 ± 67	0.09
Body weight (kg)	74 ± 12	69 ± 14	0.06
Waist (cm)	91 ± 11	89 ± 14	0.51
BMI (kg/m^2^)	26 ± 4	25 ± 5	0.26
Total cholesterol (mg/dL)	170 ± 34	165 ± 26	0.34
Triglycerides (mg/dL)	91 ± 53	67 ± 24	0.05
HDL-cholesterol (mg/dL)	57 ± 13	62 ± 13	0.11
LDL-cholesterol (mg/dL)	95 ± 32	89 ± 21	0.33
SBP (mmHg)	130 ± 20	131 ± 16	0.69
DBP (mmHg)	74 ± 10	75 ± 10	0.81
HR (bpm)	73 ± 12	77 ± 20	0.24
CSII (%)	35	56	**0.02**
CGM (%)	42	48	0.31
Hypertension (%)	14	12	0.49
Hyperlipidemia (%)	22	7	**0.04**
Retinopathy (%)	21	17	0.37
Nephropathy (%)	9	5	0.37
Neuropathy (%)	12	14	0.43
Macroangiopathy (%)	0	0	1

Data are mean ± SD and percentage

*AT* autoimmune thyroiditis, *CD* celiac disease, *HbA1c* glycated hemoglobin, *FPG* fasting plasma glucose, *BMI* body mass index, *HDL* high-density lipoprotein, *LDL* low-density lipoprotein, *SBP* systolic blood pressure, *DBP* diastolic blood pressure, *HR* heart rate, *CSII* continuous subcutaneous insulin infusion, *CGM* continuous glucose monitoring

Common carotid IMT and brachial artery FMD were significantly different among healthy subjects and T1D patients with or without concomitant autoimmune diseases. However, the posthoc test revealed a significant difference between patients with diabetes and control subjects, while no statistically significant difference was detected between T1D patients with and without concomitant autoimmune diseases (Table 2). We have also compared IMT and FMD among healthy subjects and T1D patients with AT and patients without autoimmune diseases and the results were similar.

**Table 2:** Vascular parameters of T1D patients with and without Autoimmune Thyroiditis (AT) and Coeliac Disease (CD), and healthy control subjects

Variable	Healthy Control Subjects	T1D without AT and CD	T1D with AT and or CD	P*
Number	35	69	41	–
IMT CCA right (µ)	524 ± 98	603 ± 186	611 ± 176	**0.04**
IMT CCA left (µ)	517 ± 137	635 ± 175^	631 ± 200#	**0.004**
FMD (% change)	10.1 ± 1.9	7.9 ± 4.2^	8.8 ± 4.4	**0.02**

Data are mean ± SD

*IMT* intima media thickness, *CCA* common carotid artery, *FMD* flow mediated dilation

*ANOVA, p for trend; Tukey post-hoc test ^p < 0.004 vs. control subjects; #p < 0.02 vs. control subjects

We then compared IMT and FMD between T1D patients with and without concomitant autoimmune diseases after excluding those with hypertension, hyperlipidemia, and microvascular complications (Table 3). Again, no difference was detected except for FMD, which was significantly higher in patients with AT and or CD and without microvascular complications.

**Table 3:** Vascular parameters of T1D patients without hypertension and hyperlipidemia and microvascular complications divided according to the presence and absence of Autoimmune  Thyroiditis (AT) and Coeliac Disease (CD)

Variable	T1D without AT and CD	T1D with AT and or CD	*p*
Number	50	35	–
IMT CCA right (µ)	569 ± 134	564 ± 134	0.79
IMT CCA left (µ)	581 ± 129	574 ± 129	0.88
FMD (% change)	8.5 ± 4.1	9.7 ± 3.7	0.15
Number	49	32	–
IMT CCA right (µ)	583 ± 149	582 ± 160	0.98
IMT CCA left (µ)	602 ± 132	585 ± 152	0.59
FMD (% change)	7.7 ± 3.8	9.6 ± 4.1	**0.04**

Data are mean ± SD

*IMT* intima media thickness, *CCA* common carotid artery, *FMD* flow mediated dilation

## Discussion

The results of the present study confirm that T1D patients have reduced brachial artery FMD and increased common carotid IMT compared to healthy control subjects and demonstrate that the co-occurrence of other autoimmune diseases does not play any additional role in vascular damage.

In the present study, the prevalence of AT and CD was 31% and 7.3%, which is slightly higher than that reported in the literature [3]. The difference may be attributable to the patients’ selection site. Indeed, it is more likely that patients with multiple autoimmune diseases attend the Hospital Diabetes Care Centre.

The absence of any difference in IMT and FMD between T1D patients with and without concomitant autoimmune diseases might contrast with the hypothesis that AT and CD, sharing the chronic inflammatory state with T1D, may trigger and accelerate the development of atherosclerosis [16–18]. On the contrary, we found that patients with concomitant autoimmune diseases and without microvascular complications showed higher FMD than subjects without AT and CD. The data collected in this study cannot explain this result. Still, data in the literature indicate that patients with CD on a gluten-free diet and diabetes have a lower prothrombotic state and risk of microvascular complications than T1D without CD [14]. Unfortunately, the low number of patients with T1D and CD did not allow us to perform separate analyses when evaluating FMD.

Vascular inflammation is a characteristic of atherosclerosis [16]. Recent research evaluated vascular inflammation by [(18)F]-fluorodeoxyglucose-positron emission tomography and endothelial function by the FMD technique. The authors reported that inflammation is significantly and inversely associated with endothelial dysfunction, supporting the hypothesis that a chronic inflammatory state promotes early atherosclerosis [19].

The data of the present study seem to be at odds with those of the literature since we found no worsening of markers of early atherosclerosis in T1D patients with concomitant autoimmune diseases. However, AT seems to be responsible for endothelial dysfunction if subclinical hypothyroidism and obesity coexist [20, 21], and patients with CD show early atherosclerosis if not on a gluten-free diet [22]. In our study, patients with AT were non-obese and euthyroid. Those in hormone replacement therapy were well controlled. Patients with CD were on a gluten-free diet, and the level of autoantibodies to tissue transglutaminase was below the accepted threshold. In another study, the simultaneous occurrence of diabetes and euthyroid AT did not cause a worsening of endothelial function compared with patients with diabetes alone [10].

It might be argued that the measurement of inflammatory molecules might identify subjects at high risk of developing atherosclerosis when multiple autoimmune diseases occur. Even if we did not measure these molecules, we could hypothesize that the degree of inflammation was similar in the two groups because CD patients were on a gluten-free diet and hypothyroid patients were on hormone replacement therapy.

The present study was designed to provide clinical information underlying the possible increased risk of atherosclerosis, and from this point of view, the finding is straightforward. AT and CD are not associated with early atherosclerosis, at least in euthyroid T1D patients and CD patients on a gluten-free diet. It should also be emphasized that glycemic control was acceptable. Furthermore, the prevalence of patients wearing the insulin pump was higher among those with AT and CD, suggesting an overall good glycemic control. Indeed, as known, the CSII improves not only HbA1c but also increases the time spent in the target range and reduces glycemic variability, which may both affect vascular damage if not in the target [23].

It is unlikely that these findings were influenced by sample size. The sample size was calculated assuming at least a 30% difference in FMD between the two groups. Although this value is arbitrary, it guarantees a clinically relevant difference and certainly exceeds 1 SD of FMD in control subjects. Furthermore, the number of patients enrolled was the highest in studies of this type, and the results did not even show a trend toward different vascular impairment between the two groups. The exclusion of subjects with hypertension or hyperlipidemia did not substantially alter the results. Only excluding patients with microangiopathy caused a statistically significant difference but to the advantage of patients with concomitant autoimmune diseases.

In conclusion, the data of this study confirm early vascular changes in patients with type 1 diabetes but do not show any additional damage caused by concomitant autoimmune diseases such as AT and CD. While treating patients with T1D requires utmost care, it seems unnecessary to intensify treatment in T1D patients with concomitant autoimmune diseases.

## Data Availability

The datasets used and/or analyzed during the current study are available from the corresponding author on reasonable request.
